# Analysis of potential risk factors associated with COVID-19 and hospitalization

**DOI:** 10.3389/fpubh.2022.921953

**Published:** 2022-08-05

**Authors:** Abdul-Hakeem Moazi Alharbi, Syed Imam Rabbani, Ashraf Abdel Halim Mohamed, Basil Khalid Almushayti, Nasser Ibrahim Aldhwayan, Ali Tami Almohaimeed, Abdullah Abdulrhman Alharbi, Naif Saad Alharbi, Syed Mohammed Basheeruddin Asdaq, Abdulhakeem S. Alamri, Walaa F. Alsanie, Majid Alhomrani

**Affiliations:** ^1^College of Pharmacy, Qassim University, Buraydah, Saudi Arabia; ^2^Department of Pharmacology and Toxicology, College of Pharmacy, Qassim University, Buraydah, Saudi Arabia; ^3^Consultant Pulmonologist, Buraidah Central Hospital, Buraidah, Saudi Arabia; ^4^Department of Pulmonary Medicine, Zagazig University, Zagazig, Egypt; ^5^Department of Pharmacy Practice, College of Pharmacy, AlMaarefa University, Riyadh, Saudi Arabia; ^6^Department of Clinical Laboratory Sciences, Faculty of Applied Medical Sciences, Taif University, Taif, Saudi Arabia; ^7^Centre of Biomedical Sciences Research (CBSR), Deanship of Scientific Research, Taif University, Taif, Saudi Arabia

**Keywords:** COVID-19, risk factors, complications, hospitalization, medications

## Abstract

Coronavirus disease 2019 (COVID-19) was found to cause complications in certain groups of people, leading to hospitalization. Several factors have been linked to this, such as gender, age, comorbidity, and race. Understanding the precise reasons for the COVID-19-induced complications might help in designing strategies to minimize hospitalization. A retrospective, cross-sectional observational study was conducted for patients in a COVID-19-designated specialty hospital after obtaining ethical clearance. Patients' demographic and clinical characteristics, such as age, gender, race, vaccinated status, complications, comorbidities, and medications, were retrieved from the hospital medical database. The data were statistically analyzed to determine the association between the predictors and the outcomes of COVID-19. An odds ratio (both unadjusted and adjusted) analysis was carried out to determine the risk factors for hospitalization [non-intensive care (non-ICU) and intensive care (ICU)] due to COVID-19. The data from the study indicated that the majority of patients hospitalized due to COVID-19 were male (>55%), aged > 60 years (>40%), married (>80%), and unvaccinated (>71%). The common symptoms, complications, comorbidities, and medications were fever, pneumonia, hypertension, and prednisolone, respectively. Male gender, patients older than 60 years, unemployed, unvaccinated, complicated, and comorbid patients had an odds ratio of more than 2 and were found to be significantly (*p* < 0.05) higher in ICU admission. In addition, administration of prednisolone and remdesivir was found to significantly reduce (*p* < 0.05) the odds ratio in ICU patients. The analysis of the data suggested that male gender, age above 60 years, and unvaccinated with comorbidities increased the complications and resulted in hospitalization, including ICU admission. Hypertension and type 2 diabetes associated with obesity as metabolic syndrome could be considered one of the major risk factors. Preventive strategies need to be directed toward these risk factors to reduce the complications, as well as hospitalization to defeat the COVID-19 pandemic.

## Introduction

Coronavirus disease (COVID-19) is a highly infectious illness caused by severe acute respiratory syndrome coronavirus-2 (SARS-CoV-2). The virus is genetically related to the Middle East respiratory syndrome virus and SARS-CoV-1 (1). The infection is mainly transmitted by the inhalation of droplets from infected people. The virus enters the host through inhalation of air contaminated with infected patients' sneezes, coughs, and speech (2). Touching unhygienic surfaces and the eyes, nose, or mouth of infected people could also transmit the virus to a healthy individual (3).

The first case of COVID-19 was reported in Wuhan city of China in December 2019. The infection spread to other parts of the world rapidly, and the infection reached every corner of the globe very quickly (4). Currently, the virus has infected millions of people, causing mortality in 2–3% of the world. To date, no specific therapeutic intervention has been found to treat the infection. Several classes of drugs are used to treat the symptoms, and they are mostly patient-specific (5). Vaccination is one of the most reliable approaches to building herd immunity in a population. However, due to frequent mutation of viruses and insufficient data on the precise duration of protection offered by vaccines, the efficacy of the vaccines is under elaborate study (6).

The first case of COVID-19 was reported in Saudi Arabia in March 2020. The country immediately took proactive measures, such as the closure of international borders, schools, and public places, and implemented strict precautionary measures such as wearing masks, avoiding crowded gatherings, social distancing, and mass screening of the public (7). The country is the second most affected, with more than 5.44 million confirmed cases. The mortality rate was reported to be 1–2% (8).

According to the literature, COVID-19 causes mild to moderate symptoms, such as fever, headache, body pain, and sore throat, in most individuals. Other symptoms such as loss of taste/smell, difficulty in breathing, and diarrhea were also reported (2). However, in a few people, the infection leads to severe pneumonia, congestion, hypoxia, and respiratory failure. Several factors have been reported for the occurrence of these complications due to COVID-19 (3).

The most important reasons for COVID-19-related complications are reported to be the quantum of viral exposure, host immune response, age, and comorbid conditions of the patients. A study conducted in the past suggested that 39.3% of the Saudi population suffers from different types of metabolic diseases, such as type 2 diabetes mellitus and cardiovascular diseases (9). These risk factors were found to vary from region to region and between races (10). Identifying the precise cause of hospitalization might provide an opportunity to analyze the severity and may help in proactive measures to prevent it (11). Hence, this study aimed to evaluate the factors responsible for hospitalization due to COVID-19 during the first wave of infection in a COVID-19 specialty hospital in Saudi Arabia.

## Materials and methods

### Data collection

Data were collected from a COVID-19 specialty hospital in the Qassim province of Saudi Arabia designated to treat in-patients diagnosed with COVID-19. An 11-month record (March 2020–January 2021) of in-patients admitted to the hospital was randomly retrieved after approval from the concerned authorities. All patients, irrespective of gender, age, and nationality, admitted to the COVID-19 hospital [non-intensive care (non-ICU) and intensive care (ICU)] were analyzed. Sampling of the data was performed in the duration that corresponds to the first wave of infection in the country when therapeutic interventions had limited options and were mostly carried out depending on patients' condition.

### Ethical clearance

The study was conducted after obtaining ethical clearance from the regional ethics committee of Qassim province. H-04-Q-001 is the number of the ethical clearance letter. A duly filled form with the research proposal, a letter from the institution, and a list of investigators was submitted for obtaining the approval. Prior to this, permission from the specialty hospital designated for treating COVID-19 in Qassim province was obtained for conducting the study using their recorded data. All the information about the patients was recorded as anonymous, maintaining the secrecy of their identification.

### Inclusion and exclusion criteria

All patients with COVID-19-positive results who were admitted for treatment of complications and had complete information on the predictors were included in the study, while vice versa was considered the exclusion criteria.

### Study design and participants

For this retrospective study, 619 patients' records (non-ICU = 369 and ICU = 250 patients) with confirmed COVID-19 were retrieved. All patients were diagnosed as COVID-19 positive with a real-time PCR assay for SARS-CoV-2 RNA, which analyzed genetic sequences that matched COVID-19, and then the infection was confirmed with SARS-CoV-2. The patients were clinically diagnosed as well, based on typical manifestations such as fever, cough, and respiratory distress, accompanied by chest radiological examinations (12). All 619 COVID-19 patients were considered eligible for the present study based on the inclusion criteria, and data of 39 patients were rejected, mainly due to a lack of sufficient information in their records. The medical records of each COVID-19 hospitalized patient were analyzed by the members of the research team to determine the predictors and outcome of the disease. The data of hospitalization and the mortality data (if any) with the duration of stay in the hospital were recorded for each patient during the study period. The following variables were considered for this study (13).

#### Patient and hospital characteristics

The demographic characteristics of the patients, such as gender, nationality, age, marital and employment status, were recorded for each hospitalized patient receiving treatment for COVID-19. The hospital visit information, such as date of admission, type of hospitalization (non-ICU and ICU), and discharge disposition, were also recorded.

#### Clinical characteristics

The clinical characteristics of the hospitalized COVID-19 patients, such as vaccinated status, important symptoms of disease, comorbidities (hypertension, type 2 diabetes, heart failure, chronic pulmonary disease, coronary artery disease, and cancer), and complications of COVID-19 (pneumonia, septic shock, and multi-organ failure) were recorded.

#### Pharmacological therapies

The frequently used medical interventions for treating the complications of COVID-19 were recorded. The medical records of the hospitalized patients revealed the following medications: prednisolone, favipiravir, ivermectin, hydroxychloroquine, azithromycin, and remdesivir.

#### Clinical outcomes

The clinical outcomes assessed in the COVID-19 patients included in-hospital mortality, ICU admission, and total hospital length of stay, including in ICU. The prevalence of ICU admissions can be referred to as the percentage of COVID-19 patients who had ICU admission during their stay in the hospital. On the other hand, in-hospital mortality means the percentage of COVID-19-related deaths in the hospital during the course of treatment.

#### Severity score of mortality due to COVID-19

The severity score designed by the World Health Organization was used to predict the mortality outcome in the patients hospitalized to either non-ICU or ICU (14). Different scores between 0 and 10 (“0” for uninfected and “10” for death) were assigned depending on the severity of the COVID-19-induced complications. Patients with scores of 0–3 were considered at “low” risk for COVID-19, those with scores 4–7 were indicated as “moderate” risk, and those with scores above 7 were considered to be at “high” risk of mortality. Final scores were calculated by multiplying with the number of patients presented with that particular severity of the disease (recorded as scores), and then the percentage was determined for each severity and represented in **Figure 2**.

### Statistical analysis

All the data are recorded in an Excel sheet and are represented in the form of figures and tables. A descriptive analysis of the data was carried out to determine the demographic characteristics, hospital characteristics, clinical characteristics, medications used, and clinical outcomes after treatment (survival vs. death) (15). The statistical analysis of the data was carried out using IBM SPSS 21.0 software. The categorical variables were expressed as frequencies or percentages, while continuous variables were recorded as mean values. When the data were normally distributed, the mean values were compared between groups using one-way ANOVA, and when it was not, the Mann–Whitney test was used for analysis. The chi-square test was used to calculate the odds ratio (OR), and it represented the association between potential risk factors and hospitalization. OR values suggest the odds that an outcome occurs due to an exposure compared to the outcome that is due to the absence of that particular exposure. Depending on the OR values, it is possible to study the incidences of outcome (OR < 1 indicates decreased occurrences of an event and OR > 1 indicates increased occurrences of an event). The influence of confounding factors on the analysis was corrected by evaluating the OR in the unadjusted and adjusted setups. The odds ratio values in an unadjusted and adjusted setups assessed the influence of multiple confounders or one specific confounder on the outcome of COVID-19 hospitalization, respectively (15, 16). *P* < 0.05 was considered to indicate the significance.

## Results

### Demographic characteristics of hospitalized COVID-19 patients

The demographic characteristics of hospitalized COVID-19 patients are represented in Table 1. Male patients were found to be more prevalent in both non-ICU (56.4%) and ICU (68.8%) hospitalizations than female patients. In terms of nationality, Saudis were found to be more (55.1% in non-ICU and 61.6% in ICU patients). In the age-group distribution, those older than 60 years were found to be more in both non-ICU (43.1%) and ICU (57.6%). The hospitalization of married people was found to be more (81.6% in non-ICU and 92.4% in ICU patients) than the unmarried population. The comparative data of employment status suggested that the unemployed population was found to be the most frequent hospitalized patients due to COVID-19 (59.6% in non-ICU and 63.2% in ICU) compared to employed people.

**Table 1:** Demographic characteristics of hospitalized COVID-19 patients.

**Demographic** **characteristic**	**Non-ICU patients** **(*****n*** = **369)**	**ICU patients** **(*****n*** = **250)**
**Gender**		
Male	208 (56.4)	172 (68.8)
Female	161 (43.6)	78 (31.2)
**Nationality**		
Saudis	203 (55.1)	154 (61.6)
Non-Saudis	166 (44.9)	96 (38.4)
**Age (Yrs)**		
0–20	31 (8.4)	8 (3.2)
21–40	77 (20.9)	27 (10.8)
41–60	102 (27.6)	71 (28.4)
Above 60	159 (43.1)	144 (57.6)
**Marital status**		
Married	301 (81.6)	231 (92.4)
Single	68 (18.4)	19 (7.6)
**Employment status**		
Employed	149 (40.4)	92 (36.8)
Unemployed	220 (59.6)	158 (63.2)

*Values are represented as total number (%)*.

### Clinical characteristics of hospitalized COVID-19 patients

The clinical characteristics data of COVID-19 hospitalized patients indicated that the majority of them had not received vaccines (71.8% in non-ICU and 85.2% in ICU). The most frequent symptom recorded in non-ICU patients was fever (77.2%), followed by fatigue (61.2%) and loss of smell or taste (56.6%). Also in the ICU patients, the three common symptoms were fatigue (91.6%), dyspnea (89.2%), and fever (80.8%). In non-ICU patients, the most common complication was pneumonia (4.6%), while in the ICU, patients had pneumonia (88.4%), followed by multiorgan failure (58.4%) and septic shock (35.6%). Hypertension was the most common comorbidity among both non-ICU (46.8%) and ICU (78.8%) patients, followed by diabetes (36.9% in non-ICU) and chronic pulmonary disease (62.4% in ICU) patients. Prednisolone was the most frequently used medication to manage the complications of COVID-19 in both non-ICU (60.4%) and ICU (86.8%) patients, followed by favipiravir (5.7% in non-ICU and 26.4% in ICU) (Table 2).

**Table 2:** Clinical characteristics of hospitalized COVID-19 patients.

**Clinical characteristic**	**Non-ICU patients** **(*****n*** = **369)**	**ICU patients** **(*****n*** = **250)**
**Vaccine status**		
Vaccinated	104 (28.2)	37 (14.8)
Unvaccinated	265 (71.8)	213 (85.2)
**Symptoms**		
Cough	133 (36.0)	160 (64)
Loss of smell/taste	209 (56.6)	179 (71.6)
Fever	287 (77.2)	202 (80.8)
Loss of appetite	149 (40.4)	182 (72.8)
Fatigue	226 (61.2)	229 (91.6)
Diarrhea	113 (30.6)	146 (58.4)
Vomiting	92 (24.9)	168 (67.2)
Dyspnoea	126 (34.3)	223 (89.2)
**Complications**		
Pneumonia	17 (4.6)	221 (88.4)
Septic shock	3 (0.8)	89 (35.6)
Multiorgan failure	0	146 (58.4)
**Comorbidities**		
Hypertension	173 (46.8)	197 (78.8)
Type-2 diabetes mellitus	136 (36.9)	156 (62.4)
Heart failure	11 (2.9)	71 (28.4)
Chronic pulmonary disease	104 (28.2)	163 (65.2)
Coronary artery disease	5 (1.3)	23 (9.2)
Cancer	2 (0.5)	15 (6.0)
**Medications**		
Prednisolone	223 (60.4)	217 (86.8)
Favipravir	21 (5.7)	66 (26.4)
Ivermectin	4 (1.1)	31 (12.4)
Hydroxychloroquine	6 (1.6)	42 (16.8)
Azithromycin	5 (1.3)	63 (25.2)
Remdesivir	3 (0.8)	20 (8.0)

*Values are represented as total number (%)*.

**Figure 1:**
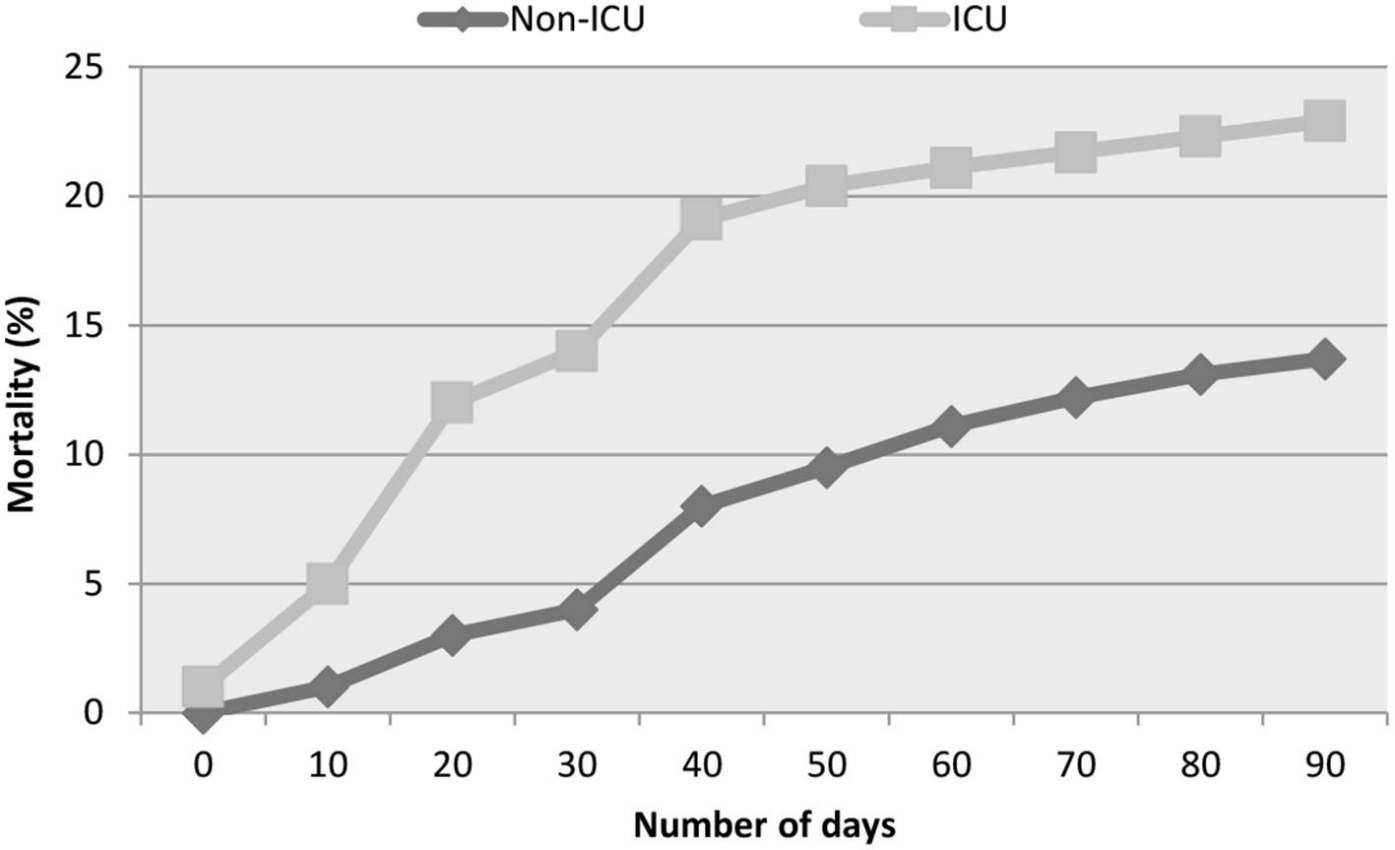
All-cause mortality recorded in COVID-19 patients.

The percentage of mortality recorded for COVID-19 patients at different intervals of days is represented in Figure 1. In non-ICU patients, the data indicated that 1% mortality was observed between 0 and 10 days of hospitalization, and this increased to 8% between 10 and 40 days. Thereafter, the percentage mostly remained steady and remained between 9.5% (50 days) and 13.7% (90 days). In the COVID-19 ICU patients, 1% of mortality was recorded on the day of hospitalization, which increased to 12% between 10 and 20 days and 19.1% between 30 and 40 days. After 50 days, the slope of the curve indicated a steady rate of mortality of 20.4% (50 days) and 22.9% (90 days).

### Association between potential risk factors and COVID-19 based on demographic characteristics

The association between the demographic characteristics and hospitalization in COVID-19 patients is given in Table 3. The unadjusted odd ratio of non-ICU male patients was found to be 3.36, and the value increased significantly (*p* = 0.03) to 5.01 in ICU hospitalization. The adjusted odds ratio also showed a significant (*p* = 0.02) increase in the ICU male patients when compared with the non-ICU hospitalization. In the female patients, although the unadjusted odds ratio was above 3, non-significant variation was observed between non-ICU and ICU hospitalization. Similarly, the adjusted odds ratio showed non-significant variation between the non-ICU and ICU female patients.

**Table 3:** Association between potential risk factors and COVID-19 hospitalization depending on demographic characteristics.

**Demographic characteristic**	**Unadjusted Odds Ratio**			**Adjusted Odds Ratio**		
	**(95% CI)**			**(95% CI)**		
	**Non-ICU**	**ICU**	***p*** **-value**	**Non-ICU**	**ICU**	***p*** **-value**
**Gender**						
Male	3.36 (2.74–4.69)	5.01 (3.14–6.68)	0.03	1.21 (0.91–1.97)	2.95 (2.05–3.32)	0.02
Female	3.02 (2.31–3.56)	3.26 (2.14–3.89)	1.06	1.26 (0.79–1.32)	1.92 (1.70–2.06)	0.11
**Nationality**						
Saudis	2.62 (1.88–2.95)	2.08 (2.01–2.24)	0.24	1.89 (0.98–2.29)	3.21 (2.62–3.74)	0.01
Non-Saudis	1.92 (1.65–2.20)	2.17 (1.21–2.92)	0.09	0.98 (0.62–1.02)	1.29 (0.71–1.78)	0.33
**Age (Yrs)**						
0–20	0.31 (0.28–0.44)	0.06 (0.01–0.09)	1.12	0.07 (0.03–1.02)	0.03 (0.01–0.05)	1.02
21–40	1.72 (1.25–1.96)	0.92 (0.61–1.16)	0.04	0.81 (0.46–0.98)	0.61 (0.21–0.88)	0.66
41–60	2.42 (1.72–2.36)	2.89 (2.44–3.48)	0.13	1.47 (0.82–1.86)	2.73 (1.88–2.52)	0.02
Above 60	3.26 (2.92–3.62)	5.16 (3.67–5.89)	0.01	2.18 (2.06–2.96)	4.06 (3.91–4.12)	0.03
**Marital status**						
Married	2.16 (1.82–2.41)	1.68 (1.34–1.81)	0.26	1.30 (0.96–1.88)	3.05 (2.96–4.48)	0.04
Single	1.77 (0.93–2.21)	1.65 (0.88–1.86)	1.33	0.85 (0.62–0.96)	1.09 (0.84–1.26)	0.63
**Employment status**						
Employed	1.91 (0.92–2.30)	1.32 (0.88–1.50)	0.16	1.10 (0.91–1.30)	0.85 (0.69–1.02)	0.56
Unemployed	2.21 (2.01–2.69)	1.89 (1.16–2.06)	0.39	1.80 (0.98–2.41)	2.52 (2.12–2.69)	0.96

*Statistical analysis: Chi-square test. Bold values indicates the ‘statistical significant values'*.

The analysis of data depending on patients' nationality indicated a significant increase (*p* = 0.01) in the adjusted odds ratio of ICU patients when compared with non-ICU hospitalization. Other values in this domain did not show any significant variation when compared between them. In the age criteria, a significant (*p* = 0.02) higher adjusted odds ratio was observed for ICU patients aged 41–60 years when compared with non-ICU hospitalization. Furthermore, patients older than 60 years had a significantly higher odds ratio when comparing non-ICU and ICU hospitalization in both unadjusted (*p* = 0.01) and adjusted (*p* = 0.03) setups. The odds ratio showed a gradual increase as the age of the patients increased but was found to be non-significant when non-ICU and ICU data were compared.

The marital status parameter indicated a significant (*p* = 0.04) increase in the adjusted odds ratio for ICU patients when compared with non-ICU for the married patients. The comparison of data between non-ICU and ICU for both unadjusted and adjusted odds ratios of unmarried/single patients did not show significant variation. Furthermore, the employment status of the hospitalized patients showed non-significant variation between non-ICU and ICU odds ratios in both unadjusted and adjusted testing modules.

### Clinical characteristics-based association between potential risk factors and COVID-19 hospitalization

The clinical analysis of vaccinated and unvaccinated patients indicated a significant (*p* = 0.04) increase in the unadjusted odds ratio for ICU hospitalization compared to non-ICU patients. A similar significant (*p* = 0.03) increase was observed for the adjusted odds ratio for ICU hospitalization when compared with non-ICU patients. In both unadjusted and adjusted odds ratios, the values for unvaccinated non-ICU and ICU patients were found to be above 3. The comparison of the non-ICU- and ICU-hospitalized unadjusted odds ratios indicated a significant (*p* = 0.02) increase, while in the adjusted odds ratio, no significant variation was observed. On the other hand, comparison of data for both unadjusted (*p* = 0.04) and adjusted (*p* = 0.01) odds ratios indicated a significant increase in ICU-hospitalized patients compared to non-ICU patients.

The three complications recorded in the hospitalized COVID-19 patients, such as pneumonia, septic shock, and multiorgan failure, indicated an odds ratio above 2 for non-ICU patients in both unadjusted and adjusted analyses. These values increased above 3 and were found to be significantly (*p* < 0.05) high for the ICU-hospitalized patients upon comparison with non-ICU patients for all the three complications. In the comorbidity conditions, hypertension and chronic pulmonary disease, the unadjusted odds ratio of ICU hospitalization increased significantly (*p* < 0.05) compared to non-ICU patients. However, the adjusted analysis indicated a significant increase in the ICU odds ratio for hypertension (*p* = 0.01), type 2 diabetes (*p* = 0.03), heart failure (*p* = 0.02), chronic pulmonary disease (*p* = 0.02), and coronary artery disease (*p* =0.04) compared with non-ICU patients.

The available data suggested that different medications, such as prednisolone, favipiravir, ivermectin, hydroxychloroquine, azithromycin, and remdesivir, were used to treat the symptoms and complications of COVID-19. Among them, the unadjusted analysis indicated a significant (*p* = 0.04) reduction in the odds ratio with remdesivir in ICU patients compared to non-ICU hospitalization. In addition to remdesivir (*p* = 0.03), the treatment of prednisolone also significantly reduced (*p* = 0.04) the odds ratio in ICU patients when compared to non-ICU hospitalization (Table 4).

**Table 4:** Association between potential risk factors and COVID-19 hospitalization depending on clinical characteristics.

**Clinical characteristic**	**Unadjusted Odds Ratio**			**Adjusted Odds Ratio**		
	**(95% CI)**			**(95% CI)**		
	**Non-ICU**	**ICU**	***p*** **-value**	**Non-ICU**	**ICU**	***p*** **-value**
**Vaccine status**						
Vaccinated	0.62 (0.38–0.74)	0.48 (0.46–0.96)	0.09	0.32 (0.28–0.59)	0.25 (0.19–0.42)	0.39
Unvaccinated	3.06 (2.98–3.12)	4.39 (2.41–4.87)	0.04	3.17 (2.66–3.41)	4.06 (2.99–4.62)	0.03
**Symptoms**						
Cough	0.74 (0.56–0.98)	0.88 (0.71–0.91)	0.09	0.81 (0.94–2.52)	0.96 (0.96–1.25)	0.11
Loss of smell/taste	2.69 (2.14–2.92)	2.03 (2.84–4.46)	1.03	2.11 (1.91–2.36)	1.86 (2.76–3.43)	0.84
Fever	0.72 (0.62–0.97)	1.14 (0.95–1.65)	0.46	1.05 (0.86–1.23)	1.15 (0.88–1.29)	0.09
Loss of appetite	0.86 (0.78–0.97)	0.78 (0.62–0.89)	0.96	1.14 (0.90–1.36)	0.96 (0.72–1.08)	0.61
Fatigue	1.22 (1.16–1.59)	2.96 (0.79–1.09)	0.02	1.89 (1.49–1.96)	1.20 (1.12–1.30)	0.07
Diarrhea	0.99 (0.81–1.32)	1.16 (0.74–1.69)	0.36	1.32 (0.72–1.53)	0.96 (0.88–1.21)	0.48
Vomiting	0.56 (0.41–0.79)	1.01 (0.89–1.23)	0.60	1.01 (0.89–1.14)	1.06 (0.82–1.28)	0.19
Dyspnoea	2.15 (2.02–2.46)	3.69 (3.15–3.96)	0.04	1.49 (1.16–1.40)	4.15 (3.92–4.41)	0.01
**Complications**						
Pneumonia	1.69 (1.14–2.63)	3.96 (2.96–4.23)	0.03	1.36 (1.11–1.59)	4.41 (2.72 −4.95)	0.02
Septic shock	1.29 (1.04–1.90)	4.06 (3.26–4.51)	0.02	1.12 (0.86–1.24)	3.97 (3.78–5.26)	0.01
Multiorgan failure	0.89 (0.44–1.02)	3.78 (2.66–3.95)	0.04	0.69 (0.42–1.16)	4.16 (2.99–4.46)	0.03
**Comorbidities**						
Hypertension	2.26 (1.92–2.58)	3.96 (2.89–4.28)	0.04	2.89 (2.79–3.16)	5.04 (3.32–5.26)	0.01
Type-2 diabetes	2.76 (2.14–2.86)	3.45 (2.36–3.12)	0.08	3.02 (2.91–3.21)	3.92 (3.06–4.09)	0.03
Heart failure	2.09 (0.92 −2.36)	2.25 (2.16–2.68)	0.06	2.16 (1.42–2.89)	3.23 (3.15–3.76)	0.02
Chronic pulmonary disease	2.62 (2.22–2.89)	3.88 (3.11–3.96)	0.03	2.82 (2.52–3.03)	3.74 (3.25–3.79)	0.02
Coronary artery disease	2.41 (2.10–2.78)	2.88 (2.52–3.23)	0.56	2.41 (2.16–2.88)	3.68 (3.39–3.86)	0.04
Cancer	1.81 (0.92–1.97)	1.21 (0.96–1.46)	0.82	1.15 (0.99–1.29)	1.07 (1.02–1.62)	0.41
**Medications**						
Prednisolone	1.66 (0.72–1.85)	1.84 (0.75–1.92)	0.07	1.44 (0.56–1.29)	0.71 (0.82–1.18)	0.04
Favipravir	1.41 (0.71–1.12)	1.27 (0.63–1.59)	0.08	0.99 (0.52–1.34)	1.01 (0.37–1.50)	0.14
Ivermectin	2.54 (2.32–2.74)	3.14 (2.81–3.69)	0.19	3.14 (2.62–3.79)	3.78 (3.39–4.68)	0.48
Hydroxychloroquine	2.09 (0.66–2.81)	1.81 (1.29–3.54)	0.07	2.51 (2.39–2.68)	2.33 (2.19 −2.46)	0.19
Azithromycin	3.69 (3.47–3.80)	3.58 (3.02–3.66)	0.26	2.63 (2.44–2.79)	3.12 (2.96–3.56)	0.36
Remdesivir	0.72 (0.59–0.91)	0.41 (0.39–0.57)	0.04	0.56 (0.34–0.68)	0.30 (0.28–0.42)	0.03

*Statistical analysis: Chi-square test. Bold values indicates the ‘statistical significant values'*.

### Severity score of mortality in hospitalized COVID-19 patients

**Figure 2:**
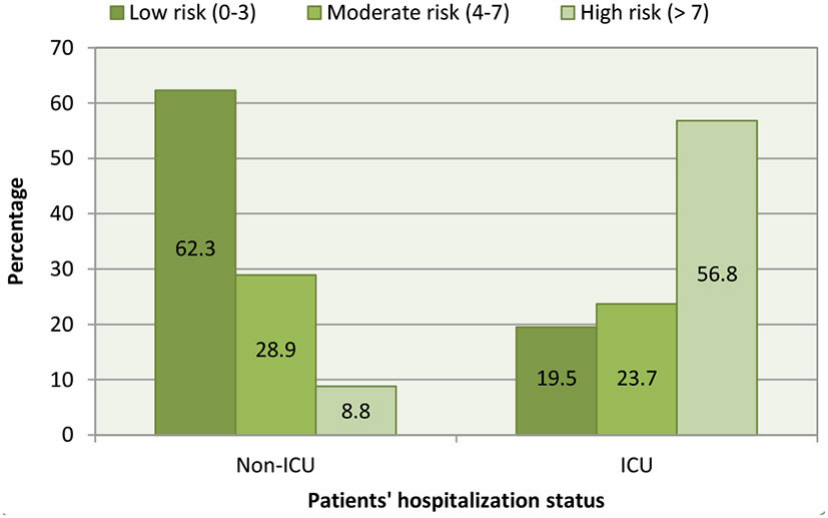
Severity score in hospitalized patients due to COVID-19.

Figure 2 represents the percentage of severity scores due to COVID-19 in the hospitalized patients. Of the non-ICU patients, 62.3% were found to be at low risk, 28.9% at moderate risk, and 8.8% at high risk of mortality due to COVID-19. The analysis of ICU patients revealed that 56.8% of them are at high risk, 23.7% at moderate, and 19.5% are at low risk of COVID-19-induced mortality.

## Discussion

The present study assessed the potential risk factors of hospitalization due to COVID-19. The analysis of the data indicated that the hospitalization of male patients was found to be more in both non-ICU (56.4%) and ICU (68.6%) admissions than that of female patients (Table 1). The unadjusted odds ratio (OR) increased for male patients severalfold and was found to be 3.36 for non-ICU patients, which further increased to 5.01 for ICU admission. The comparison of these two values indicated a significant (*p* = 0.03) increase for ICU-hospitalized patients. In addition, the adjusted odds ratio of ICU admission was found to be significantly (*p* = 0.02) higher than that of non-ICU patients (Table 3). The observations suggested that male gender could be a risk factor for hospitalization, including ICU due to COVID-19. A similar observation was found in an earlier study where the male population was found to be at higher risk of not only the disease but also hospitalization due to complications (16). The Y-chromosome has been implicated as the risk factor for COVID-19-induced complications (17).

The risk of hospitalization was found to be higher among Saudi nationals than among non-Saudis in both non-ICU (55.1%) and ICU (61.6%) admissions (Table 1). The analysis to determine the potential risk indicated that Saudi nationals are slightly at a higher risk of hospitalization than other nationals, as observed in both the unadjusted and adjusted odds ratios. Their chances of complications due to COVID-19 and admission to ICU (OR = 3.21) were also found to be higher than those for non-Saudis (OR = 1.29). The comparative analysis revealed that the ICU admission of Saudi nationals showed a significantly (*p* = 0.01) higher odds ratio (3.21) than non-ICU (OR = 1.89) in the adjusted analysis (Table 3). Earlier studies indicated that a significant portion of the Saudi population (39.3%) suffers from several metabolic diseases, such as type 2 diabetes, hypertension, and obesity (18). Occurrences of metabolic diseases were found in the population in the early age. Obesity which was considered to be one of the risk factors of metabolic diseases was found to be prevalent among 18% of Saudi children and rises to 39.9% among adolescents (19). The incidences of metabolic diseases are increasing at an alarming rate in the Saudi population and are reported to be due to lack of physical activity, changing life style, and shift from traditional diet to those rich in carbohydrates, fats, and carbonated beverages (9). There are multiple pathways, such as altered immunological response, blood circulation, and inflammatory processes, reported for the COVID-19-induced complications in the patients suffering from metabolic diseases (20). Since these comorbidities act as potential risks of COVID-19-induced complications, a higher prevalence of hospitalization could be linked to this in the present study (21).

A higher prevalence of metabolic diseases and chronic disorders can also be observed in the clinical characteristics of hospitalized patients (Table 2). Except for cancer, these comorbidities showed an odds ratio above 2 for both non-ICU- and ICU-hospitalized patients. Moreover, a significantly (*p* < 0.05) higher OR was found for ICU patients than for non-ICU patients (Table 4). Furthermore, the association of advanced age, comorbidities, and an increased risk of COVID-19-induced complications is reported in the literature. The data from the present study suggest that the increase in OD is directly proportional to the age of the patients. The OR values for older adults were found to be more than 2, indicating the enhanced risk associated with COVID-19. A significantly (*p* < 0.05) higher OR (> 4) in ICU patients older than 60 years supports the association of age, comorbidities, and potential risk of hospitalization due to COVID-19 (18–22).

Comorbidities are known to complicate COVID-19 through multiple mechanisms, such as dysfunction of renin–angiotensin, coagulatory, circulatory, and immunological systems (23). Furthermore, the marital status and higher incidences of OR could indicate that elderly patients with multiple diseases are at risk of COVID-19-induced complications. In addition, marital status may increase the chance of viral transmission due to close contact among family members (24). Studies have indicated that marital status could play both positive and negative influence on the anxiety and stress that was experienced during the COVID-19 pandemic (25). Quarantine and self-isolation methods adopted to reduce the transmission of the infection was reported to affect the mental health adversely and increased the chances of COVID-19-associated complications (26). Furthermore, the non-significant increase in OD (>1.80) observed in unemployed patients can also be linked to patients older than 60 years (Table 3).

The vaccinated status of the COVID-19 patients indicated that most of the non-ICU (71.8%) and ICU (85.2%) admissions had not received the required dosages of approved vaccines (Table 1). A significantly higher odds ratio was observed for unvaccinated ICU (OR > 3) patients when unadjusted (*p* = 0.04) and adjusted (*p* = 0.03) analyses were carried out (Table 2). The findings suggest that the vaccinated status might protect the population from the complications of COVID-19. Saudi Arabia has approved major COVID-19 vaccines, such as AstraZeneca, Pfizer, Moderna, and Johnson (27). Currently, the vaccination status in the country has crossed 90% (28). Since the WHO has approved a few vaccines and prioritized the recipient groups, only limited numbers were vaccinated during the study period.

The most common symptoms of non-ICU- and ICU-hospitalized patients were found to be fever (77.2%) and fatigue (91.6%), respectively (Table 1). The unadjusted odds ratio analysis indicated a significantly (*p* = 0.02) higher value for ICU patients (OR = 2.96) than for non-ICU patients (OR = 1.22). In addition, dyspnea in both unadjusted (*p* = 0.04) and adjusted (*p* = 0.01) odds ratios was found to be higher for ICU (OR > 3.6) than for non-ICU (OR = 1.49–2.15) patients (Table 4). Fatigue could be due to desaturation of blood oxygen levels, and dyspnea is one of the frequent symptoms associated with respiratory distress (29). Both these symptoms are reported to occur when the body's immune system responds aggressively to COVID-19. The cytokine storm reported during this phase of immunological response causes pneumonia and interferes with respiratory function (30). The appearance of complications, such as pneumonia, septic shock, and multi-organ failure, could also be the consequence of the immunological reaction due to COVID-19 (Tables 2, 4). The significant (*p* < 0.05) elevation of the OR (>4) in ICU patients (Table 4) is in agreement with the previous studies where pneumonia, septic shock, and multi-organ failure were considered the major reasons for ICU hospitalization (31).

The medication analysis revealed that prednisolone was the most frequent intervention in both non-ICU-hospitalized (60.4%) and ICU-hospitalized (86.6%) patients (Table 2). Some of the other medications also reduced the odds ratio, but significant variation was observed only with prednisolone and remdesivir. Prednisolone in the adjusted analysis decreased significantly (*p* = 0.04) the odds ratio of ICU patients compared to non-ICU patients. In both unadjusted and adjusted analyses, remdesivir significantly reduced the ICU odds ratio significantly (*p* < 0.05) compared to non-ICU hospitalization (Table 4). Prednisolone is a corticosteroid reported to be effective in reducing the inflammatory process associated with COVID-19. The drug was approved by the WHO for treating mild to moderate complications of COVID-19. The intravenous administration of prednisolone was found to be effective in reducing the actions of pro-inflammatory mediators and the hyper-immunological responses during COVID-19 (32). The drug was first authorized for emergency use by the U.S. FDA to treat complicated COVID-19 cases. The drug exhibits its action by inhibiting SARS-CoV-2 RNA-dependent RNA polymerase, essential for viral replication (33). The findings of the present study are in line with those of the previous research where both prednisolone and remdesivir were found to be effective in reducing the complications associated with COVID-19 (32, 33). Favipiravir and azithromycin were also frequently used in the management of COVID-19-related complications, but these medications did not show significant variation in OR values in both non-ICU and ICU patients (Table 4).

The percentage of mortality recorded among the non-ICU patients indicated a progressive increase as the number of days in the hospital increased. The highest percentage of mortality in non-ICU patients was found to be between 30 and 50 days. On the other hand, the mortality percentage increased rapidly for ICU-admitted patients from the 10th to the 40th day (Figure 1). These findings support previous research, indicating that mortality due to COVID-19 in ICU hospitalization was highest between the 10th and the 40th day (3) of comorbidities. Advanced age, male gender, and unvaccinated status could all be the major factors for the observed hospitalized COVID-19 patients (34).

Analysis of severity score in the hospitalized patients due to COVID-19 indicated that 8.8% of non-ICU admissions are at higher risk of mortality, while 62.3% of them were found to be at lower risk. On the other hand, 56.8% of ICU-admitted patients were considered at higher risk of mortality due to the complications of COVID-19, and the lower risk in them was found to be 19.5% (Figure 2). As reported in the literature, advanced age and presence of several comorbidities along with lack of immunization could be the reasons for higher risk in patients admitted to the ICU (10).

The prevalence and mortality in the first wave of COVID-19 in Saudi Arabia were reported to be 6.1 and 2.0%, respectively. During this phase, where limited options were available for treating the COVID-19-induced complications, the healthcare professionals were reported to follow the latest guidelines of the World Health Organization. These include several therapeutic interventions listed in Table 2 and mechanism ventilation (invasive and non-invasive) as well as intubation. These options were attempted depending on the severity of COVID-19-induced complications. The research conducted on these patients suggested that mortality was associated with leukocytosis, anemia, thrombocytopenia, and higher levels of prothrombin time, troponin, and ferritin (35). In addition, the study indicated that such abnormal biomarker levels and higher incidences of mortality were frequent among the aged COVID-19 patients diagnosed with metabolic diseases (36).

The findings of the study on the group of COVID-19 patients represented an important analysis of factors responsible for the hospitalization. As per the available data, a significant proportion of Saudi population suffers from different types of metabolic syndrome, including type 2 diabetes, hypertension, and obesity (19). Several studies in the past have linked the presence of metabolic syndrome with COVID-19-induced complications (19). Obesity is considered to be important metabolic syndrome that has been linked to several diseases, such as hypertension, type 2 diabetes, and coronary artery disease. A study conducted in the past indicated that the risk of hypertension increased up to 70% in obese patients (37). The prevalence of type 2 diabetes in Saudi population was reported to increase to 38.9% in the obese population (38). The analysis of the data of the present study data suggested higher odd ratios for hypertension (OD = 5.04) and type 2 diabetes (OD = 3.92) for the ICU-hospitalized patients due to COVID-19 (Table 4). This information suggested that obesity, which is common in Saudi population, could be one of the major risk factors for the COVID-19-induced complications and hospitalization. The data from this study could be used by healthcare providers to target a specific group of the population in designing strategies to reduce the severity of any future diseases depending on the risk associated with them.

### Limitation of study

The present study represents the analysis of COVID-19-hospitalized patients when the country experienced the first wave of infection. The study was conducted by retrieving the patients' data from a COVID-19-designated specialty hospital. Several advancements have occurred since then in medical interventions and the vaccinated status of the population, which is reported to have crossed over 90%. Hence, the findings will only represent the data from a select group of patients during the study period and may not reflect the whole population affected by COVID-19 in the region.

## Conclusion

In the present study, the analysis of the data indicated several confounders for the hospitalization of COVID-19 patients. Gender, age, vaccinated status, dyspnea, comorbidities, and complications due to COVID-19 were found to be the major risk factors for hospitalization, including ICU admission. Obesity, which is common in Saudi population, could be one of the important risk factors for the COVID-19-induced complications and hospitalization. Since coronavirus mutates at regular intervals with increasing virulence, healthcare providers must investigate these factors and prioritize the preventive strategies to minimize the risk of hospitalization for any future outbreak of the disease.

## Data availability statement

The original contributions presented in the study are included in the article/supplementary material, further inquiries can be directed to the corresponding author/s.

## Ethics statement

The studies involving human participants were reviewed and approved by Regional Ethics Committee of Qassim province, H-04-Q-001. The Ethics Committee waived the requirement of written informed consent for participation.

## Author contributions

Under the supervision of SR, A-HA, AH, BA, NAld, AAlm, AAlh, and NAlh carried out the research methodology. AAla was responsible for formal analysis of the work, while WFA, A-HA, AH, and BA participated in writing the original draft of the manuscript. MA administered the project. SAs was instrumental in reviewing and editing the manuscript. All authors contributed to the article and approved the submitted version.

## Funding

This study was supported by Taif University [No. TURSP (2020/257)]. AlMaarefa University in Riyadh, Saudi Arabia, assisted SAs with this research (TUMA-2021-1).

## Conflict of interest

The authors declare that the research was conducted in the absence of any commercial or financial relationships that could be construed as a potential conflict of interest.

## Publisher's note

All claims expressed in this article are solely those of the authors and do not necessarily represent those of their affiliated organizations, or those of the publisher, the editors and the reviewers. Any product that may be evaluated in this article, or claim that may be made by its manufacturer, is not guaranteed or endorsed by the publisher.
